# Sexual Dysfunction Induced by Antidepressants—A Pharmacovigilance Study Using Data from VigiBase^TM^

**DOI:** 10.3390/ph17070826

**Published:** 2024-06-24

**Authors:** Rene Zeiss, Kathrin Malejko, Bernhard Connemann, Maximilian Gahr, Verena Durner, Heiko Graf

**Affiliations:** 1 Department of Psychiatry and Psychotherapy III, University of Ulm, Leimgrubenweg 12-14, 89075 Ulm, Germany; 2 Department of Psychiatry and Psychosomatic Medicine, Städtisches Klinikum Karlsruhe, Kaiserallee 10, 76133 Karlsruhe, Germany

**Keywords:** antidepressant-induced sexual dysfunction, pharmacovigilance, disproportionality analysis, adverse drug reaction

## Abstract

Sexual dysfunction is a common side effect of antidepressants, significantly impacting patients’ quality of life and treatment adherence. This study investigates the relationship between sexual dysfunction and antidepressants by analyzing data from VigiBase™, the World Health Organization’s global database of individual case safety reports. In this study, we examined, for the first time, reports related to sexual response—desire, arousal, and orgasm—by grouping appropriate side effect terms and calculated the reporting odds ratios (RORs) for various antidepressants. The findings of this study highlight a high disproportional reporting of sexual dysfunction, particularly with selective serotonin reuptake inhibitors and serotonin–norepinephrine reuptake inhibitors. In contrast, agents such as agomelatine, bupropion, and mirtazapine showed a lower association. Furthermore, we investigated the correlation between reporting odds ratios and the binding affinities of antidepressants to specific neurotransmitter receptors and transporters, unveiling significant relationships that provide insights into the pharmacodynamic pathways underlying these adverse effects. For instance, a positive correlation was observed between the serotonin transporter and side effects in the category desire: r (19) = 0.67, *p* = 0.001 These insights underscore the necessity for clinicians to consider sexual side effects when prescribing antidepressants and to monitor and address these issues to improve patient outcomes.

## 1. Introduction

Major depressive disorder (MDD) is one of the largest contributors to the global burden of disease and one of the top ten factors increasing the burden [1]. With an annual prevalence of about 4 to 5% and an estimated lifetime prevalence of almost 20%, MDD is not only disabling but also very common [2]. Besides psychotherapy, antidepressants (ADs) are one of the most effective treatment options for the management and relapse prevention of MDD [3,4,5]. Since the discovery of tricyclic antidepressants in the 1950s, several other classes of ADs are available and widely prescribed, namely, selective serotonin reuptake inhibitors (SSRIs), serotonin–norepinephrine reuptake inhibitors (SNRIs), and other ADs such as mirtazapine and vortioxetine. As there appear to be no significant differences in efficacy between the ‘older’ and ‘newer’ substances [6], individual patient preferences, comorbidities, and side-effect profiles are relevant considerations when prescribing types of ADs. Despite their good tolerability in general, ADs still exert side effects and adverse drug reactions (ADRs) that are often transient but, in some cases, serious and life-threatening or even life-limiting [7]. ADRs associated with ADs range from gastrointestinal symptoms, cardiac dysfunction, hepatotoxicity, weight gain, central nervous system disorders, and bleeding events to sexual dysfunction and other causes [7]. 

Despite that low sexual desire and functioning has been reported in 25 to 75% of patients with MDD who are not taking medication [8,9], numerous studies reported a prevalence of sexual dysfunction across different ADs, ranging from 4 up to 80% [10,11,12]. Since SSRIs/SNRIs are one of the most prescribed ADs, it is not surprising that several studies report an incidence of sexual dysfunction ranging from 58 to 73% during treatment with these medications [13]. This high prevalence underpins the critical need for a deeper understanding of sexual dysfunction under ADs in clinical practice [14,15]. Here, it is of note, that this side effect does not only considerably impact quality of life, it also affects treatment adherence [7,16,17], and about 20% of patients discontinue antidepressive medication due to the inability to have an orgasm and lost sexual interest [18]. Considering that regular intake is recommended for months or even longer, this side effect has relevant implications on relapse frequency and prevention. 

A large body of literature suggests that ADs affect sexual functioning in various aspects and involves one or more phases of the sexual response cycle [10,19,20,21], with delayed ejaculation as the most commonly reported. Further studies describe primarily delayed or absent orgasms and a lack of sexual desire and/or arousal (for a review, see the following: [10]). However, despite the multitude of studies on this topic, it is not reliably possible to draw precise conclusions about which phases of the sexual response cycle are primarily affected by ADs. This may be due to methodological flaws regarding study design and measurements on the one hand and, on the other hand, to the observation that rates and/or the severity of sexual dysfunction differs widely among the different ADs investigated, presumably owing to their receptor profile and binding affinity. 

Although AD-related sexual dysfunction is a well-known common side effect that has been investigated in various studies, the underlying mechanisms still remain poorly understood. Considering the mechanism of action of ADs and the modulation of human sexual activity by various neurotransmitters and hormones, it seems plausible that AD-related alterations in neuromodulator levels seem to play a pivotal role. In particular, an increase in serotonergic neurotransmission is accompanied by a diminished neurofunctional activation within the human reward system and in brain regions relevant for emotion processing, leading to attenuated sexual interest and arousal. Other neuromodulators, such as dopamine, exert facilitating effects on sexual functioning (for a review, see the following: [22]) Nevertheless, it is of note that each of the neuromodulators and hormones may act in different ways on different phases of the sexual response cycle and further through different neuronal systems and interactions in the control of sexual functioning.

To further elucidate the association between sexual dysfunction and several AD classes with varying alterations of neuromodulators, we conducted a study using a large pharmacovigilance database to provide a broader understanding and real-world impact of these medications. The primary aim of this study is to elucidate the impact of ADs on sexual dysfunction, with a specific focus on the different phases of the sexual response cycle such as sexual desire, arousal, and orgasm. This investigation seeks to bridge the gap in current research by employing a comprehensive pharmacovigilance analysis of data from VigiBase™, the WHO’s global database of individual case safety reports (ICSRs). This study aims to explore the complex pharmacodynamic interactions underlying antidepressant use and sexual dysfunction. By analyzing how the binding affinity of antidepressants to specific neurotransmitter receptors affects sexual health, we aim to shed light on the mechanistic pathways driving these adverse effects.

## 2. Results

### 2.1. Number of Individual Case Safety Report (ICSRs)

On the index date of 1 April 2020 (database status at the time of query), the total number of ICSRs in VigiBase is 22,288,675. A total of 27,128 ICSRs were associated with the desire category, 37,631 with the arousal category, 16,691 with the orgasm category, and 9745 with sexual dysfunction. 

### 2.2. Signals and Reporting Odds Ratios (RORs)

**Figure 1:**
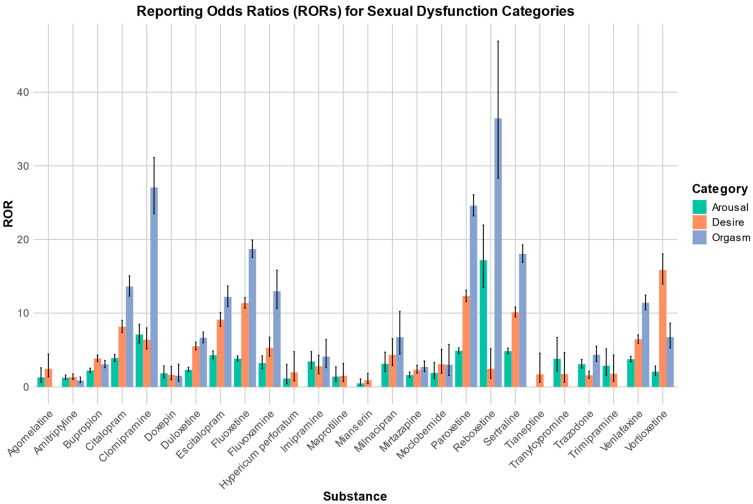
Reporting odds ratios for the categories of arousal, desire, and orgasm, along with their respective 95% confidence intervals.

A summary of the RORs and their respective 95% confidence intervals for each category, along with the signals found, are presented in Table 1. The signals identified are presented in bold. Figure 1 shows a plot with the RORs for the substances investigated and for the three stages “desire”, “arousal”, and “orgasm”.

### 2.3. Association between RORs and Binding Affinity 

#### 2.3.1. Desire

The Pearson correlation showed a positive relationship between the RORs in the desire category and an affinity for the SERT: r (19) = 0.67, *p* = 0.001. There was also a negative Pearson correlation between the RORs and an affinity for the H1 receptor: r (10) = −0.92, *p* =< 0.0001. Negative Pearson correlations were also found between the RORs and an affinity for 5HT2B, 5HT2c, and a1. 5HT2B: r (8) = −0.84, *p* = 0.003; 5HT2c: r (11) = −0.60, *p* = 0.031; a1: r (4) = −0.85, *p* = 0.032. 

#### 2.3.2. Arousal

In the arousal category, we found a negative Pearson correlation between the RORs and H1: r (10) = −0.59, *p* = 0.045.

#### 2.3.3. Orgasm

There was no correlation between the RORs in the orgasm category and the affinities for the receptors in question.

#### 2.3.4. Sexual Dysfunction

In the sexual dysfunction subgroup, a negative Pearson correlation was found between the RORs and 5HT2B, 5HT2c, a1, and H1. 5HT2B: r (6) = −0.8, *p* = 0.017; 5HT2c: r (9) = −0.75, *p* = 0.0075; a1: r (2) = −0.98, *p* = 0.016; H1: r (7) = −0.81, *p* = 0.008.

**Figure 2:**
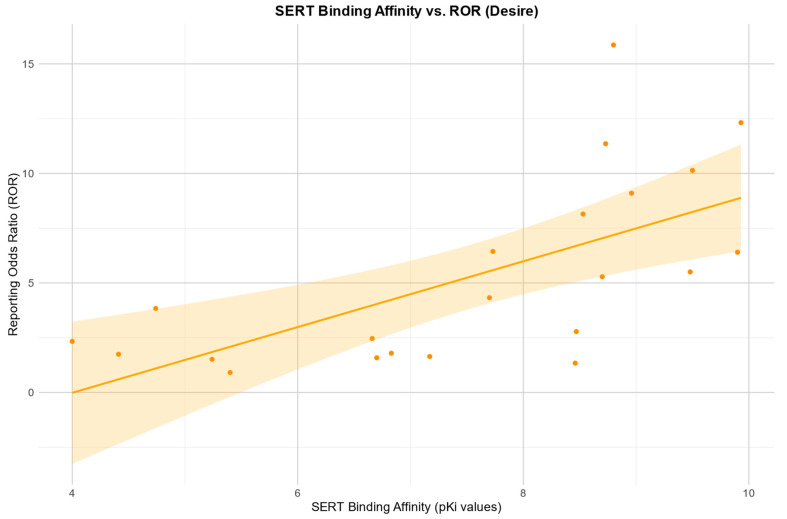
Correlation between the reporting odds ratios (RORs) for the category “desire” and the affinity measured by the pKi for the serotonin transporter (SERT), including the 95% confidence interval and a linear regression line.

**Figure 3:**
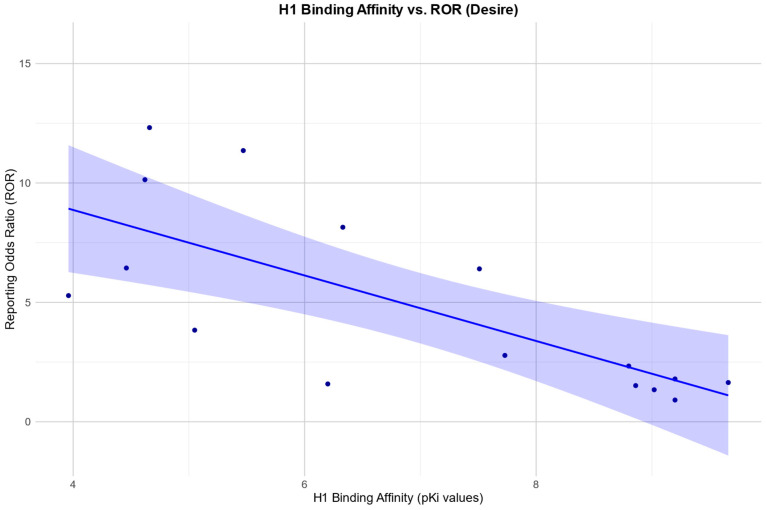
Correlation between the reporting odds ratios (RORs) for the category “desire” and the affinity measured by the pKi for the histamine H1 receptor (H1), including the 95% confidence interval and a linear regression line.

**Figure 4:**
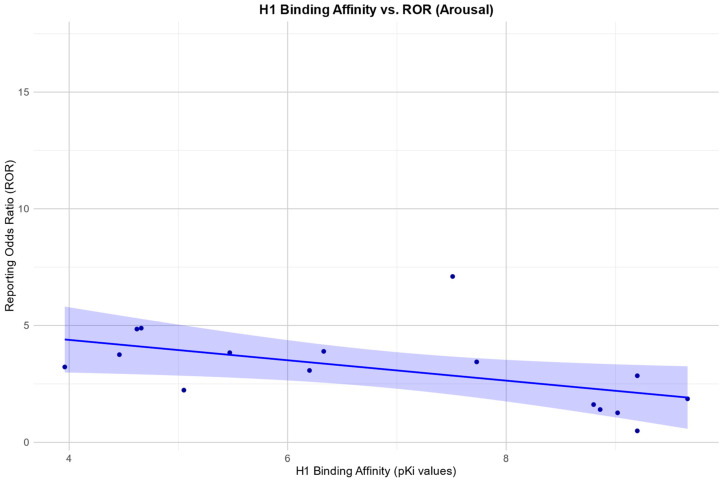
Correlation between the reporting odds ratios (RORs) for the category “arousal” and the affinity measured by the pKi for the histamine H1 receptor (H1), including the 95% confidence interval and a linear regression line.

Scatter plots for the associations found between the RORs in the desire category and an affinity for the SERT and H1, respectively, as well as the association found between the RORs in the arousal category and an affinity for the H1 receptor, are depicted in Figure 2, Figure 3 and Figure 4.

## 3. Discussion

Our study confirmed the well-known association of sexual dysfunction related to pharmacological antidepressant therapy in pharmacovigilance databases, and we observed significant signals in all three main domains (desire, arousal, and orgasm) of the sexual response cycle for almost all of the investigated ADs, especially for SSRIs and SNRIs. In accordance with the literature, fewer signals were found for substances that do not act by inhibiting the monoamine reuptake. 

Our observation of higher rates of sexual dysfunction under serotonergic agents are in line with a systematic review and network meta-analysis that also reported higher rates of sexual dysfunction for antidepressants compared with a placebo, especially for SSRIs and SNRIs [23]. The lowest rates were observed for trazodone and vortioxetine, although they were also higher than those observed for the placebo. The drugs identified by us as having a low risk of affecting the various stages of sexual function, namely, agomelatine, bupropion, and mirtazapine, were not included in the aforementioned study [23].

Previous attempts to investigate sexual dysfunction in relation to ADRs with the help of pharmacovigilance databases have been few and far between. Trenque et al. conducted an analysis of French pharmacovigilance data. They found several signals for SRIs and sexual dysfunction but none for escitalopram and duloxetine. They concluded that sexual dysfunction is particularly vulnerable to under-reporting, because it is a topic that both patients and carers are reluctant to discuss and report [24]. We agree with the view of Trenque et al. that underreporting in this area is a significant problem. In our study, this was particularly evident in the reports received on female patients, some of which represent only a small fraction of the total number of reports on sexual dysfunctions. In order to avoid undermining the primary focus of this study, namely, the pharmacodynamic–pharmacoepidemiological aspect, separation according to gender was omitted in our final evaluation. In view of the current literature, both male and female patients appear to be strongly affected by sexual dysfunction under antidepressants. The low rate of reports of affected women is particularly surprising in light of the fact that sexual dysfunction, as a symptom of a depressive disorder, appears to have an even higher prevalence in women [8]. It is therefore recommended that increased reporting of behavior and regular queries about adverse ADRs in daily clinical practice be encouraged. In a descriptive study of drug-induced sexual dysfunction, Valeiro et al. analyzed data from the national spontaneous reporting database of the Netherlands [25]. This revealed a similar pattern, with an unusually high representation of cases for male patients. In addition, the results of our study are consistent with those of the study bei Valeiro et al., which indicated that antidepressants, especially SSRIs, were associated with a high disproportional reporting regarding sexual adverse effects [25].

Fewer signals were found for substances that do not act by inhibiting the monoamine reuptake. This was especially true for agomelatine, bupropion, and mirtazapine [26]. In contrast to recently published studies, our study also showed signals for vortioxetine, which also acts partly via serotonin reuptake inhibition. One possible contributor for the high RORs for vortioxetine might be an issue described in the literature: There might be an increase in the reporting of adverse effects for a substance shortly after its market launch [27]. This phenomenon, characterized by an increase in reports shortly after introduction and a decrease after some time, especially of relatively “mild” side effects, was first described by Weber in 1984 and is therefore known as the “Weber effect” [28]. Nevertheless, the question of whether this pattern is reflected in today’s reported behavior and in today’s databases is the subject of a controversial debate [29]. Nonetheless, the signals for vortioxetine should receive attention, and the effects on sexuality should be investigated in future work. 

In a recent work by Gordijn et al., ADRs reported online by patients on a dedicated platform were studied. For sexual dysfunction, a share of 50% was found for antidepressant substances. In line with the results in our study, disorders of libido (desire) were reported in particular. The signals found by Gordijn et al. also fit the results in our analysis, so mirtazapine, bupropion, and amitriptyline were not significant. For the investigated SSRIs, a signal was found in each case. A difference is shown for vortioxetine, which, however, had few users at the time of the study and therefore can only be investigated to a limited extent [30].

Regarding the pharmacodynamic aspects of our study, there are several points to discuss. The pharmacodynamic approach of our work showed a correlation between an affinity for the SERT and the occurrence of disorders in “desire”. This is consistent with previous research, which found an increased risk of sexual dysfunction for SSRIs in particular [31,32]. There is also emerging evidence of an increased risk of a condition called post-SSRI sexual dysfunction associated with serotonergic antidepressants [33]. Although our study design does not allow us to draw conclusions about this condition, we also found an association between an affinity for the SERT and the occurrence of sexual dysfunction. It is difficult to capture the described problem in studies with spontaneous reporting systems, but our work could provide an opportunity to consider the degree of serotonin reuptake inhibition with regard to post-SSRI sexual dysfunction in future studies. 

Further evidence for the role of the serotonin system in the development of sexual ADRs is the negative correlation between calculated RORs and the affinity for an effect at various serotonin receptors for desire and sexual dysfunction [34,35,36]. For instance, our data show lower RORs in the category of desire for substances with a high affinity and an antagonistic effect at 5-HT2c receptors like mirtazapine or amitriptyline. This is consistent with some studies that have found a reduction in side effects regarding movement disorders with SSRIs when a 5-HT2c antagonist is added [37].

For arousal and desire, there was a negative correlation between the RORs and H1 receptor affinity, suggesting a ‘protective effect’ for an antagonistic effect at the H1 receptor. The role of histamine in sexual function is not fully understood. There’s some literature on its role in male sexual function, as histamine may act as a modulator in Leydig cells and therefore in testosterone synthesis [38]. Furthermore, there are data suggesting a role of histamine in human penile erection, suggesting that H1-receptor antagonism can potentiate histamine-induced relaxation in the corpus cavernosum and neurally mediated corporal relaxation [39,40]. However, as antidepressants with an affinity for the H1 receptor often also have a high affinity for certain serotonin receptors, it is difficult to make precise statements about the relationship with our data.

Our study showed a negative correlation between the RORs and an affinity for the a1 receptor. The compounds studied have antagonistic activity at the a1 receptor. However, for several reasons, our results allow for only limited conclusions regarding the role of the a1 receptor. Only a few of the compounds in this study have a relevant effect on the adrenergic system via antagonism at the a1 receptor. At the same time, the substances in question often have a pronounced effect on various serotonin receptors. As discussed above, these are likely to play a crucial role in sexual function. However, further evaluation of this issue using pharmacoepidemiological and pharmacodynamic approaches would require additional studies and study designs.

It should be noted that our study has certain limitations that require further investigation in this area. For the evaluation, we only had information on the occurrence of ADRs associated with a specific substance. The influence of the underlying disease or polypharmacy could therefore not be taken into account. Since the investigated substances are predominantly used for the treatment of depression, we assume that the underlying disease should have had an influence on all investigated substances. The data available to us did not permit any more detailed analyses of the influencing factor of the age of the patients concerned. However, from the available literature, it can be said that sexual dysfunction as an ADR is a problem that can affect patients of any age category. Age, per se, should also not make a relevant difference with regard to pathophysiology and the receptors/transporters involved.

A major weakness common in pharmacovigilance studies performed with ADR databases is underreporting, and it is known that only a mere fraction of all ADRs occurring is reported [41,42]. Underreporting is a relevant problem, especially for ADRs in connection with sexual dysfunction, but it also affects pivotal studies, in particular and not exclusively, postmarketing analyses. Furthermore, underreporting should affect all substances in a similar way. As previously stated, the findings of our study indicate that there were signs of an underreporting bias regarding sexual dysfunction, particularly among female patients. This made it challenging to conduct a gender-specific analysis.

Furthermore, because there is no information on the number of patients exposed to a particular substance, the data do not allow for a relative or absolute risk to be estimated. This limitation also makes it impossible to infer a causal relationship, although there is support for a relationship between the degree of disproportionality and actual risk [43].

As several PTs have been grouped into categories that represent different aspects of sexuality, the data do not allow for any conclusions to be drawn about the severity of the sexual dysfunction but only about the aspect that is affected. For example, a loss of libido and a decrease in libido are part of the same category. 

Although the large number of adverse drug reactions reported in VigiBase is a major strength of this analysis, it is also necessarily a weakness, since the information comes from a variety of sources around the world and the likelihood that the suspected adverse drug reaction is related to a medicine may not be the same in all cases.

In summary, our study demonstrates a high disproportional reporting of sexual dysfunction for the majority of antidepressants investigated, particularly those with a high rate of SERT inhibition. It can be observed that substances such as agomelatine, bupropion, and mirtazapine demonstrate a lower associated risk. Nevertheless, there seems to be significant underreporting, particularly among female patients. Our findings emphasize the necessity of monitoring and addressing sexual side effects in clinical practice, with the aim of enhancing patient adherence and quality of life. By examining the relationship between RORs and the binding affinities of antidepressants to various neurotransmitter receptors, this study provides valuable insights into the pharmacodynamic mechanisms underlying antidepressant-induced sexual dysfunction. These insights particularly highlight the degree of SERT inhibition and suggest an insufficiently understood role of the histamine system. Such information can inform future research and guide the development of clinical strategies to mitigate these adverse effects.

## 4. Materials and Methods

We conducted a pharmacovigilance analysis using VigiBase™, the WHO’s global database of individual case safety reports (ICSRs). VigiBase^TM^ is the world largest world’s largest repository of ADR reports. The period covered in this study was 1968–2020 (04/2020). We performed a case/non-case approach for calculating the ROR. The use of RORs is a proven method for carrying out disproportionality analyses [44,45]. They are calculated in a similar way to the odds ratios in case-control studies. In the case of disproportionality analyses, there is no classic “control group”, so that all affected reports represent the “cases” group and all other reports in the database represent the “non-cases” control group [44,46]. RORs were calculated when the number of ICSRs was ≥3 [44]. Probabilistic methods commonly used in the mentioned database were used to detect duplicate individual case safety reports.

### 4.1. Database Query and Search Strategy

ADRs in VigiBase^TM^ are classified according to the Medical Dictionary for Regulatory Activities (MedDRA), which was used to identify reports related to sexual disorders. The version of MedDRA used was version 22.1.

As there were no defined search criteria or standardized MedDRA queries (SMQs) for sexual disorders at the time of the study, several preferred terms (PTs) were grouped according to the sexual response phases introduced by Masters and Johnson’s Human Sexual Response Model [47] and Kaplan’s Triphasic Model [48] for desire, arousal, and orgasm. For desire, PT libido decreased, PT libido disorder, and PT loss of libido; for arousal, PT erectile dysfunction and PT vulvovaginal dryness; and for orgasm, PT anorgasmia, PT ejaculation disorder, PT ejaculation failure, PT female orgasmic disorder, PT male orgasmic disorder, and PT orgasm abnormal. Furthermore, PT sexual dysfunction, PT female sexual dysfunction, and PT male sexual dysfunction were analyzed as a separate category. All MedDRA terms were screened and categorized independently by RZ and HG, and disagreements were resolved by a discussion. A case was defined as an ICSR associated with one of the PTs of interest mentioned above, and all other reports were defined as non-cases. 

### 4.2. Antidepressant-Exposure

AD-exposure was defined as a mentioning of the following ADs in an ICSR either as suspected/interacting or concomitant: agomelatine, amitriptyline, bupropion, citalopram, clomipramine, doxepin, duloxetine, escitalopram fluoxetine, fluvoxamine, hypericum perforatum, imipramine, maprotiline, mianserin, milnacipran, mirtazapine, moclobemide, paroxetine, reboxetine, sertraline, tianeptine, tranylcypromine, trazodone, trimipramine, venlafaxine, and vortioxetine. The classification of antidepressants in this study was based on their widespread use and availability, ensuring that the analysis reflects the experiences of a diverse patient population in different healthcare settings.

### 4.3. Pharmacodynamic Aspect

A univariate linear regression analysis was used to assess the effect of a compound’s affinity for specific targets and sexual dysfunction. The Pearson correlation coefficient was calculated to measure the correlation between the RORs and the respective pKi of the compound for the serotonin transporter (SERT), dopamine transporter (DAT), norepinephrine transporter (NET), 5-HT1a receptor (5HT1a), 5-HT2a receptor (5HT2a), 5-HT2b receptor (5HT2b), 5-HT2C receptor (5HT2c), adrenergic receptor α1 (a1), adrenergic receptor α2 (a2), and histamine H1 receptor (H1). The pKi values used are the geometric mean of the values determined in competition binding assays performed on human targets. The database used to extract information about the ligand affinity was The Guide to PHARMACOLOGY and ChEMBL database [49,50]. Substances with an affinity for the target of pKi of <4 were excluded from the analysis. 

Since the data retrieved from VigiBase^TM^ are anonymized and cannot be related to an individual person, ethics approval was not required. The information does not represent the opinion of the UMC or the World Health Organization.

Data analysis and graphical work was performed with “R”, a language and environment for statistical computing (R Foundation for Statistical Computing, Vienna, Austria; URL, https://www.R-project.org/). R version 4.2.0

## 5. Conclusions

In conclusion, our article shows that case–non-case analyses can be used to investigate the clinically significant issue of sexual dysfunction in antidepressant treatment. Several agents, such as agomelatine, bupropion, mirtazapine, and also amitriptyline, appear to have a significantly lower risk of sexual dysfunction than SSRIs or SNRIs. In addition, the combined pharmacoepidemiological–pharmacodynamic approach shows that several transmitter systems are involved. While a high affinity for the SERT seems to be associated with an increase in sexual dysfunction, particularly in terms of desire, a high affinity for certain serotonin receptors, like 5-HT2c, with an antagonistic effect seems to be associated with a lower risk for sexual dysfunction. Furthermore, the histamine system appears to play a role that is not yet fully understood. Our findings indicate that substances with an antagonistic effect and a high affinity for the H1 receptor may be associated with a lower risk for sexual side effects. Finally, our work is a possible new approach to investigating complex side effect areas, such as sexual dysfunction, by grouping appropriate preferred terms to gain new insights into the mechanism of action.

## Figures and Tables

**Table 1:** Reporting odds ratios and the respective 95% confidence interval for the categories “desire”, “arousal”, “orgasm”, and “sexual dysfunction”. Signals identified are presented in bold font. A signal is defined as a lower 95% confidence interval greater than 1. NA = no data available or number of individual case safety reports is <3.

Reporting Odds Ratios incl. 95% Confidence Interval				
	Desire	Arousal	Orgasm	Sexual Dysfunction
Substance	ROR (95% CI)	ROR (95% CI)	ROR (95% CI)	ROR (95% CI)
Agomelatine	**2.46 (1.36–4.44)**	1.29 (0.64–2.57)	NA	**4.35 (2.07–9.14)**
Amitriptyline	**1.34 (1.02–1.76)**	**1.26 (1–1.6)**	0.88 (0.57–1.35)	1.51 (0.98–2.31)
Bupropion	**3.84 (3.43–4.29)**	**2.23 (1.97–2.53)**	**3.05 (2.6–3.58)**	**5.83 (5.01–6.8)**
Citalopram	**8.15 (7.36–9.01)**	**3.89 (3.44–4.4)**	**13.62 (12.31–15.06)**	**13.88 (12.19–15.81)**
Clomipramine	**6.4 (5.13–7.99)**	**7.1 (5.93–8.49)**	**27.06 (23.51–31.14)**	**4.94 (3.25–7.5)**
Doxepin	1.64 (0.97–2.77)	**1.86 (1.22–2.83)**	1.52 (0.76–3.05)	1.31 (0.49–3.48)
Duloxetine	**5.5 (5.01–6.05)**	**2.33 (2.07–2.64)**	**6.65 (5.96–7.43)**	**11.08 (9.9–12.41)**
Escitalopram	**9.1 (8.2–10.09)**	**4.29 (3.78–4.87)**	**12.2 (10.88–13.68)**	**20.59 (18.32–23.14)**
Fluoxetine	**11.36 (10.67–12.09)**	**3.83 (3.51–4.19)**	**18.7 (17.56–19.93)**	**7.56 (6.68–8.56)**
Fluvoxamine	**5.28 (4.15–6.73)**	**3.22 (2.48–4.19)**	**12.97 (10.64–15.82)**	**4.88 (3.21–7.43)**
Hypericum perforatum	1.98 (0.82–4.76)	1.14 (0.43–3.04)	NA	**4.41 (1.65–11.75)**
Imipramine	**2.78 (1.81–4.27)**	**3.44 (2.48–4.78)**	**4.09 (2.61–6.42)**	1.47 (0.55–3.92)
Maprotiline	1.51 (0.72–3.18)	1.4 (0.73–2.7)	NA	NA
Mianserin	0.91 (0.45–1.82)	0.49 (0.22–1.09)	NA	NA
Milnacipran	**4.33 (2.87–6.52)**	**3.12 (2.07–4.7)**	**6.73 (4.43–10.24)**	**4.7 (2.44–9.05)**
Mirtazapine	**2.33 (1.87–2.91)**	**1.62 (1.29–2.02)**	**2.69 (2.07–3.5)**	**3.95 (2.98–5.25)**
Moclobemide	**3.07 (1.85–5.09)**	**1.91 (1.11–3.3)**	**2.99 (1.55–5.75)**	2.27 (0.85–6.06)
Paroxetine	**12.32 (11.57–13.11)**	**4.88 (4.5–5.3)**	**24.6 (23.2–26.08)**	**21.78 (20.1–23.59)**
Reboxetine	**2.46 (1.17–5.17)**	**17.19 (13.46–21.96)**	**36.45 (28.31–46.93)**	**14.77 (8.89–24.55)**
Sertraline	**10.14 (9.48–10.84)**	**4.85 (4.47–5.25)**	**18.04 (16.89–19.27)**	**16.94 (15.5–18.5)**
Tianeptine	1.71 (0.64–4.55)	NA	NA	NA
Tranylcypromine	1.75 (0.65–4.66)	**3.79 (2.15–6.68)**	NA	NA
Trazodone	**1.58 (1.17–2.15)**	**3.07 (2.55–3.71)**	**4.35 (3.43–5.51)**	**2.91 (1.99–4.24)**
Trimipramine	1.79 (0.75–4.31)	**2.85 (1.57–5.15)**	NA	NA
Venlafaxine	**6.44 (5.89–7.04)**	**3.75 (3.4–4.14)**	**11.41 (10.47–12.44)**	**8.96 (7.9–10.17)**
Vortioxetine	**15.86 (13.95–18.03)**	**2.09 (1.56–2.81)**	**6.75 (5.28–8.63)**	**20.84 (17.31–25.09)**

## Data Availability

The data utilized in this study may be accessed via VigiBase^TM^ of the Uppsala Monitoring Center.
